# Falls in ED patients: do elderly patients on direct oral anticoagulants bleed less than those on vitamin K antagonists?

**DOI:** 10.1186/s13049-021-00866-6

**Published:** 2021-04-06

**Authors:** Martin Müller, Ioannis Chanias, Michael Nagler, Aristomenis K. Exadaktylos, Thomas C. Sauter

**Affiliations:** 1 Department of Emergency Medicine, Inselspital, Bern University Hospital, Bern University, Bern, Switzerland; 2 University Institute of Clinical Chemistry, Inselspital Bern University Hospital, and University of Bern, Bern University, Bern, Switzerland

**Keywords:** Anticoagulants, Bleeding, Direct oral anticoagulants, Fall, Intra cranial bleeding, Syncope, Vitamin K antagonist

## Abstract

**Background:**

Falls from standing are common in the elderly and are associated with a significant risk of bleeding. We have compared the proportional incidence of bleeding complications in patients on either direct oral anticoagulants (DOAC) or vitamin K antagonists (VKA).

**Methods:**

Our retrospective cohort study compared elderly patients (≥65 years) on DOAC or VKA oral anticoagulation who presented at the study site – a Swiss university emergency department (ED) – between 01.06.2012 and 01.07.2017 after a fall. The outcomes were the proportional incidence of any bleeding complication and its components (e.g. intracranial haemorrhage), as well as procedural and clinical parameters (length of hospital stay, admission to intensive care unit, in-hospital-mortality). Uni- and multivariable analyses were used to compare the studied outcomes.

**Results:**

In total, 1447 anticoagulated patients were included – on either VKA (*n* = 1021) or DOAC (*n* = 426). There were relatively more bleeding complications in the VKA group (*n* = 237, 23.2%) than in the DOAC group (*n* = 69, 16.2%, *p* = 0.003). The difference persisted in multivariable analysis with 0.7-fold (95% CI: 0.5–0.9, *p* = 0.014) lower odds for patients under DOAC than under VKA for presenting with any bleeding complications, and 0.6-fold (95% 0.4–0.9, *p* = 0.013) lower odds for presenting with intracranial haemorrhage. There were no significant differences in the other studied outcomes.

**Conclusions:**

Among elderly, anticoagulated patients who had fallen from standing, those under DOACs had a lower proportional incidence of bleeding complications in general and an even lower incidence of intracranial haemorrhage than in patients under VKAs.

**Supplementary Information:**

The online version contains supplementary material available at 10.1186/s13049-021-00866-6.

## Introduction

Falls are a leading cause of trauma in elderly patients presenting at the emergency department (ED) [1, 2]. One of the main type of falls are from standing and these falls are often associated with head and neck trauma [3]. About 30% of people age 65 years and older fall at least once a year [4]. Falls are responsible for loss of independence, disability, and even death in the elderly [5–7]. In addition to individual harm, falls pose an important economic burden to any healthcare system, with costs ranging between 0.9 and 1.5% of the total health care expenditure in the USA, United Kingdom, Australia, and Europe [8]. The severity of injuries after a fall increases with age [7]. The increased morbidity and mortality in older patients after a fall are partially associated with anticoagulant therapy and the resulting increased risk of bleeding complications [9]. Anticoagulant therapy is more prevalent in elderly patients, especially in patients with atrial fibrillation [10], and is intended to prevent stroke [11, 12]. Not surprisingly, it has been shown that both falls and oral anticoagulation are risk factors for the development of intracranial haemorrhage [13, 14]. Thus, in up to 50% of the patients with an indication for anticoagulation for stroke prevention, anticoagulation is not prescribed, as the perceived greater risk of bleeding – especially after falls – outweighs the perceived risk of stroke [15, 16] and a prior history of a fall is a clinically useful risk factor for poor outcome in anticoagulated elderly patients [17]. This underlines the need for trials on the interaction between falls and anticoagulation.

In recent years, DOACs have gradually replaced classical vitamin K antagonists (VKA) in anticoagulation therapy, even in special patient groups such as elderly patients [18]. In this context, it is important to examine the risk of bleeding complications in patients under different treatments for anticoagulation.

A meta-analysis of randomised controlled trials in elderly patients found no increased risk for major bleeding in patients on DOAC compared to patients on VKA [19]. A subgroup analysis from the ENGAGE AF trial found fewer bleeding complications and a reduction in short-term mortality in patients on edoxaban compared to patients on warfarin [20]. Although the data suggest that DOACs are an effective and safe alternative to VKA in elderly patients, these results must be confirmed with real-life data for all DOACs, as elderly patients in clinical trials are known to have fewer comorbidities and better adherence to medication than real-life patients, where non-adherence to medication therapy is often reported [21, 22].

Therefore, we aim to analyse our series of elderly, anticoagulated ED patients admitted after a fall and compare patients on DOAC with those on VKA with regards to bleeding complications. The primary outcome of this investigation is the proportional incidence of any bleeding complications in DOAC vs. VKA patients after falls from standing. Secondary outcomes are the proportional incidence of different types of haemorrhage (e.g. intracranial), admission to the intensive care (ICU), length of ED and hospital stay as well as in-hospital mortality.

## Methods

### Study design and setting

This retrospective cohort study was conducted at the interdisciplinary adult ED of Bern University Hospital, Inselspital, Switzerland. In this Level 1 trauma centre, about 45,000 patients from a catchment area of 2 million people are seen each year [23].

### Inclusion criteria

We included patients of at least 65 years of age on oral anticoagulants, i.e. DOAC or VKA therapy, admitted after a fall from standing for whatever reason (including syncope, trips and slipping and falls from fewer than three stairs) over the study period (01.06.2012 to 01.07.2017).

### Exclusion criteria

The exclusion criteria were i) age < 65 years, ii) no documented oral anticoagulation on ED admission, iii) no acute fall from standing as the reason for ED admission.

### Study outcomes

The primary study outcome was the proportional incidence in any documented bleeding complications - including intracranial haemorrhage (i.e. epidural haematoma, subdural haematoma, intracerebral bleeding, subarachnoid bleeding), abdominal bleeding, thoracic bleeding, arterial soft tissue haematoma, epistaxis, venous soft tissue haematoma. “Any type of bleeding” was chosen as composite outcome because any bleeding resulted in an emergency department presentation and were therefore judged to be clinically relevant.

Secondary outcomes were i) proportional incidence of specific types of bleeding, particularly intracranial haemorrhage, ii) procedural outcomes (length of hospital stay and in-hospital as well as ICU admission), and iii) clinical outcomes (in-hospital mortality).

### Data handling

The medical report included the patient’s history, a comprehensive list of diagnoses, medication intake, clinical findings, and discharge procedure and was routinely stored electronically in full text in our computerised database (E-Care, ED 2.1.3.0, Turnhout, Belgium).

**Fig. 1:**
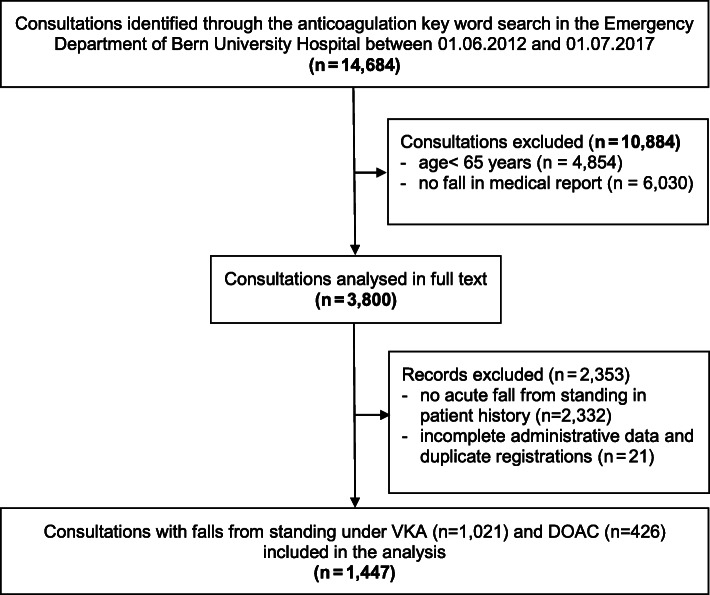
Flowchart of the study**.** Abbreviations: DOAC, direct oral anticoagulant; VKA, Vitamin K antagonist

For data extraction, we conducted a broad keyword search in the whole patient database during the study period, including all substance and brand names of oral anticoagulant drugs approved in Switzerland, together with variants in spelling and common mistakes (DOAC: apixaban, dabigatran, edoxaban, rivaroxaban, VKA: acenocoumarol, phenprocoumon and warfarin), combined with the logic operator “OR” (*n* = 14,684). In the resulting dataset, a sensitive keyword search was used to identify patients with falls, tripping, slipping, and syncope (Flowchart: Fig. 1). The medical reports of the identified patients were analysed in full text to exclude patients without a fall (including syncope) from standing or fewer than 3 stairs.

### Data extraction

Baseline characteristics were recorded, including age, sex, type and indication for anticoagulation (atrial fibrillation, mechanic valve, thromboembolic disease, thrombophilia, peripheral artery disease, and postsurgical status). Data on the fall characteristics and trauma details (ground level, number of steps fallen, syncope), bleeding complications and characteristics were also extracted from the medical record. We classified the mechanisms of the fall from standing height into three categories:

- Category one: falls from tripping or slipping from the patient’s own height (ground level);
- Category two: falls from fewer than three stairs.
- Category three: falls due to a syncope that do not have the same trauma mechanism

In addition, we manually extracted specific comorbidities of the patient (diabetes, liver disease, chronic obstructive lung disease (COPD), malignancy, congestive heart failure, past myocardial infarction, peripheral arterial disease (PAVD), past cerebrovascular insult / transient ischaemic attack, hemiplegia, dementia, renal insufficiency, connective tissue disease, alcoholism, peptic ulcer disease) and risk factors for falls and bleeding (previous falls, HAS-BLED-score) from the medical report. In addition, the group of patients were characterised by extracting laboratory values (i.e. haemoglobin, platelets, leucocytes, INR, creatinine, sodium, and potassium) from the computerised database. Therapy to inhibit platelet aggregation was also recorded.

### Handling of missing data

Clinical characteristics were coded as present if they were documented in the comprehensive medical report of the ED. No documentation of clinical characteristic was coded as not present. All covariables that were included in the multivariable models had no missing data. For the documentation of the indication of OAC, a “non-documented”-category was coded. For further characteristics, such as laboratory data (Supplement 1), the numbers (per cent) of non-missing values are shown.

### Data analysis

The analysis was performed with Stata® 13.1 (StataCorp, The College Station, Texas, USA). For descriptive analysis, categorical variables were presented as absolute numbers, accompanied by relative numbers. Continuous variables were presented as medians with interquartile ranges. The distributions in the two different study groups (DOAC vs. VKA) were compared using the chi square test (for categorical variables) or the Wilcoxon rank sum test (for continuous variables).

Multivariable regression analysis was performed to quantify the impact of the type of anticoagulation on the presentation with bleeding complications (primary or secondary outcomes) after a fall from standing. All effect sizes were presented as odds ratios (OR) or geometric mean ratios (GMR) with 95% confidence interval.

Multivariable logistic regression analysis was performed that included all variables from the HAS-BLED score and Charlson Comorbidity Index [24] that had shown at least slight evidence for an association with the type of anticoagulation (*p* < 0.2). Additionally, age, the number of medications, and number of diagnoses were incorporated into the final model.

Sensitivity analysis was based on i) a stepwise backward selection (*p* < 0.2) logistic regression model and ii) a multivariable model with adjustment for the Charlson Comorbidity Index and the HAS-BLED score. The effect of the type of anticoagulation on secondary outcomes was studied with a backward selection logistic regression model (for binary outcomes) or a linear regression model adjusted for the variables identified through univariable analysis (for continuous outcomes). In linear regression models, the outcome was ln-transformed and the coefficients expressed as exponentials, corresponding to the GMR [25].

*p* < 0.05 was set as significant. No adjustment for multiple testing was performed. There was no formal study size calculation as all ED consultations from the start of the use of the digital, comprehensive documentation in E-Care, ED 2.1.3.0, Turnhout, Belgium were included.

### Ethics

The present investigation was registered and approved by the Ethics Committee of Canton Bern, Switzerland under the number 073/2015. According to Swiss law, no informed consent is needed for the use of coded patient data.

## Results

Over the study period, 1447 anticoagulated patients aged at least 65 years presented to our ED after an acute fall from standing. Of these patients, 1021 (70.6%) were on VKA and 426 (29.4%) on DOAC therapy (Fig. 1).

### Patient characteristics

The median age was 80 (IQR 74–85) years and 50.1% of the patients were males. There was no significant difference between the treatment groups with respect to either age (*p* = 0.240) or gender (*p* = 0.681).

There were relatively more patients with previous cerebrovascular insult or transient ischemic attack (*p* = 0.001), malignancy (*p* = 0.045), dementia (*p* < 0.001), alcoholism (*p* < 0.001), peptic ulcer disease (*p* = 0.025) as well as an increased number of medications taken (*p* = 0.008) in the group of patients under DOAC. The, percentage of patients with peripheral artery disease was higher in the VKA group (Table 1).

**Table 1:** Baseline characteristics for each type of anticoagulation

	VKA (***n*** = 1021)	DOAC (***n*** = 426)	Total (***n*** = 1447)	***p***
**Demographic characteristic**				
Sex, male, [n (%)]	508 (49.8)	217 (50.9)	725 (50.1)	0.681
Age (years), med (IQR)	80 (74–85)	80 (74–85)	80 (74–85)	0.240
**Comorbidities**				
HAS-BLED Score, [med (iqr)]	3 (2–3)	3 (2–3)	3 (2–3)	0.083
Charlson Comorbidity Index, [med (iqr)]	6 (4–8)	6 (5–8)	6 (4–8)	0.015
No. of drugs, [med (iqr)]	7 (4–10)	8 (5–10)	7 (5–10)	0.008
No. of diagnoses, [med (iqr)]	5 (4–6)	5 (4–6)	5 (4–6)	0.004
Hypertension, [n (%)]	744 (72.9)	325 (76.3)	1069 (73.9)	0.177
Diabetes, [n (%)]	229 (22.4)	99 (23.2)	328 (22.7)	0.737
CHF, [n (%)]	225 (22.0)	103 (24.2)	328 (22.7)	0.375
Coronary artery disease, [n (%)]	97 (9.5)	43 (10.1)	140 (9.7)	0.728
Cerebrovascular disease,[n (%)]	228 (22.3)	131 (30.8)	359 (24.8)	0.001
Hemiplegia, [n (%)]	115 (11.3)	50 (11.7)	165 (11.4)	0.796
Renal insufficiency, [n (%)]	401 (39.3)	145 (34.0)	546 (37.7)	0.061
PAVD, [n (%)]	117 (11.5)	27 (6.3)	144 (10.0)	0.003
Liver disease, [n (%)]	17 (1.7)	13 (3.1)	30 (2.1)	0.092
COPD, [n (%)]	98 (9.6)	53 (12.4)	151 (10.4)	0.107
Malignancy, [n (%)]	104 (10.2)	59 (13.8)	163 (11.3)	0.045
Dementia, [n (%)]	106 (10.4)	73 (17.1)	179 (12.4)	< 0.001
Alcoholism, [n (%)]	8 (0.8)	14 (3.3)	22 (1.5)	< 0.001
Connective tissue disease, [n (%)]	36 (3.5)	14 (3.3)	50 (3.5)	0.820
Peptic ulcer disease, [n (%)]	174 (17.0)	94 (22.1)	268 (18.5)	0.025
AIDS, [n (%)]	0 (0.0)	0 (0.0)	0 (0.0)	–
History of bleeding, [n (%)]	177 (17.3)	78 (18.3)	255 (17.6)	0.658

*Abbreviations*: *CHF* Congestive heart failure, *COPD* Chronic obstructive pulmonary disease, *CVI* Cerebrovascular insult, *DOAC* Direct oral anticoagulant, *iqr* Interquartile range, *med* Median, *TIA* Transient ischemic attack, *PAVD* Peripheral artery disease, *VKA* Vitamin K antagonist

As regards the indications for oral anticoagulation, there were more patients with atrial fibrillation and past surgical status in the DOAC group but more patients with artificial valves in the VKA group (Table 2). Furthermore, the indication was not documented in ≥10% in both groups, with a higher percentage in the VKA group.

**Table 2:** Information on anticoagulation in the study groups

	VKA (***n*** = 1021)	DOAC (***n*** = 426)	Total (***n*** = 1447)	***p***
**Type OAC, [n (%)]**				
VKA	1021 (100.0)	0 (0.0)	1021 (70.6)	
DOAC				
Rivaroxaban	–	362 (85.0)	362 (25.0)	
Apixaban	–	44 (10.3)	44 (3.0)	
Edoxaban	–	5 (1.2)	5 (0.3)	
Dabigatran	–	15 (3.5)	15 (1.0)	< 0.001
**Indication OAC, [n (%)]**				
Atrial fibrillation	546 (53.5)	294 (69.0)	840 (58.1)	
Mechanical heart valve	34 (3.3)	3 (0.7)	37 (2.6)	
Thromboembolic event	175 (17.1)	77 (18.1)	252 (17.4)	
Post-surgery	0 (0.0)	6 (1.4)	6 (0.4)	
Other	20 (2.0)	4 (0.9)	24 (1.7)	
Not documented	246 (24.1)	42 (9.9)	288 (19.9)	< 0.001

*Abbreviation*: *DOAC* Direct oral anticoagulants, *OAC* Oral anticoagulant, *VKA* Vitamin K antagonist

The intake of antiplatelet agents and specific laboratory values are shown in Supplement 1. No significant differences were found between the two anticoagulation groups with respect to long term therapy with antiplatelet agents (*p* = 0.067) and the use of non-steroidal antirheumatic medication (*p* = 0.124). As regards the laboratory values, there was a slightly higher creatinine value in the VKA patient group (*p* = 0.006). An elevated international normalised ratio (INR) in the VKA group was as expected, because of the mechanism of action of this medication.

### Trauma details

In total, 81.3% of the patients suffered falls from ground level, 3.7% falls from stairs, and 15% were admitted after a syncope. There was no significant difference between the type of anticoagulation and the mechanism of trauma (*p* = 0.834).

### Type of bleeding and frequency in each group

There were relatively more bleeding complications in the VKA group (*n* = 237, 23.2%) than in the DOAC group (*n* = 69, 16.2%, *p* = 0.003).

The proportional incidence of intracranial haemorrhage was also slightly higher in the VKA group (15.7% versus 9.9%, *p* = 0.004).

The distribution of bleeding complications in the different study groups is shown in Table 3. The most frequent type of intracranial haemorrhage was subdural haemorrhage (VKA 48.5% vs. DOAC 36.2%). After intracranial haemorrhage, venous soft tissue haematoma was the most common bleeding type (VKA 15.6% vs. DOAC 13.0%).

**Table 3:** Type of bleeding according to type of anticoagulation in the 306 patients with documented bleeding [n (%)]

	VKA (***n*** = 237)	DOAC (***n*** = 69)	Total (***n*** = 306)
Intracranial haemorrhage	160 (67.5)	42 (60.8)	202 (66.0)
Epidural haemorrhage	4 (1.7)	1 (1.4)	5 (1.6)
Subdural haemorrhage	115 (48.5)	25 (36.2)	140 (45.8)
Intracerebral	19 (8.0)	7 (10.1)	26 (8.5)
Subarachnoid	17 (7.2)	8 (11.6)	25 (8.2)
Subarachnoid and subdural	16 (6.8)	6 (8.7)	22 (7.2)
Subdural and intracerebral	6 (2.5)	1 (1.4)	7 (2.3)
Subarachnoid and intracerebral	5 (2.1)	1 (1.4)	6 (2.0)
Abdominal bleeding	4 (1.7)	2 (2.9)	6 (2.0)
Thoracic bleeding	5 (2.1)	1 (1.4)	6 (2.0)
Arterial soft tissue haematoma	4 (1.7)	4 (5.8)	8 (2.6)
Venous soft tissue haematoma	37 (15.6)	9 (13.0)	46 (15.0)
Epistaxis	5 (2.1)	4 (5.8)	9 (2.9)

*Abbreviation*: *DOAC* Direct oral anticoagulant, *VKA* Vitamin K antagonist

### The impact of type of anticoagulation on the proportional incidence of any bleeding and the secondary outcomes

The univariable analysis for the composite primary outcome “any bleeding” and the secondary outcomes is shown in Table 4.

**Table 4:** The impact of type of anticoagulation on the primary and secondary outcomes – univariable analysis

	OR (95% CI)[DOAC vs. VKA]	***p***
Any bleeding complication	0.639 (0.476–0.859)	*0.003*
Intracranial haemorrhage	0.589 (0.410–0.844)	*0.004*
Abdominal bleeding	1.199 (0.219–6.572)	0.834
Thoracic bleeding	0.478 (0.056–4.105)	0.501
Arterial soft tissue haematoma	2.410 (0.600–9.681)	0.215
Venous soft tissue haematoma	0.574 (0.275–1.200)	0.140
Epistaxis	1.926 (0.515–7.208)	0.330
ICU admission	0.791 (0.611–1.025)	0.076
In-hospital mortality	1.186 (0.725–1.940)	0.498
	**GMR (95% CI)[DOAC vs. VKA]**	***p***
LOS hospital [days]	1.026 (0.853–1.235)	0.783

*Abbreviation*: *DOAC* Direct oral anticoagulants, *GMR* Geometric mean ratio, *ICU* Intensive care unit, *LOS* Length of stay, *OR* Odds ratio

Significant reductions (*p* = 0.003 and *p* = 0.004) of the odds for bleeding in DOAC compared to VKA patients were found for any bleeding complication and intracranial haemorrhage in the univariable analysis.

The multivariable analyses were performed to determine the impact of the type of anticoagulation on the presentation with bleeding complications after a fall from standing – including characteristics presented in Tables 1 and 2 (with *p* < 0.2). The bleeding odds ratio (DOAC vs. VKA) was 0.678 (95% CI: 0.5–0.9) see Table 5).

**Table 5:** The impact of type of anticoagulation on the presentation with bleeding complications after a fall from standing – multivariable analysis

Outcome: any bleeding	OR (95% CI)	***p***
Type of anticoagulation		
VKA	1.000 base	
DOAC	0.678 (0.498–0.924)	0.014
Age, per year increase	1.022 (1.003–1.041)	0.022
Hypertension	0.748 (0.553–0.012)	0.060
Cerebrovascular disease	0.570 (0.407–0.799)	0.001
Renal insufficiency	0.664 (0.489–0.901)	0.009
PAVD	0.775 (0.463–1.297)	0.332
Liver insufficiency	0.539 (0.158–1.840)	0.324
COPD	0.466 (0.263–0.826)	0.009
Malignancy	0.864 (0.551–1.356)	0.525
Dementia	0.890 (0.581–1.365)	0.594
Alcoholism	1.606 (0.598–4.311)	0.347
Peptic ulcer disease	1.257 (0.888–1.779)	0.197
No. of medications, per additional drug more	1.016 (0.973–1.062)	0.470
No. of diagnosis, per additional diagnosis	0.889 (0.803–0.984)	0.023

*Abbreviation*: *COPD* Chronic obstructive pulmonary disease; *DOAC* Direct oral anticoagulants, *No* Number *OR* Odds ratio, *PAVD* Peripheral artery disease, *VKA* Vitamin K antagonist

### Sensitivity analysis

A sensitivity analysis for the primary outcome of any bleeding including i) a stepwise backward selection (*p* < 0.2) logistic regression model or ii) an adjustment for the Charlson Comorbidity Index and the HAS-BLED score and iii) restricting the analysis to the *n* = 1177 patient with falls from their own height (excluding syncope and falls from stairs) revealed similar ORs of i) 0.7, 95% CI: 0.5–0.9, *p* = 0.016, ii) 0.7, 95% CI: 0.5–0.9, *p* = 0.005, iii) 0.7, 95% CI: 0.5–1.0, *p* = 0.039.

### The impact of type of anticoagulation on secondary outcomes

Taking the VKA group as baseline, we compared the secondary outcomes of intracranial haemorrhage, ICU admission, length of stay and in-hospital mortality. Apart from a higher proportional incidence of intracranial haemorrhage in the VKA group (OR: 0.63, 95% CI: 0.43–0.91, *p* = 0.013), no significant differences (*p* > 0.05, see Table 6) were found for other types of bleeding. No significant differences were found in the length of hospital stay (*p* = 0.890), ICU admission (*p* = 0.767) or in-hospital mortality (*p* = 0.751).

**Table 6:** The impact of type of anticoagulation (DOAC vs. VKA with VKA as baseline) on secondary outcomes – multivariable logistic backward selection analysis with variables shown in Table 3

**Outcome**	**OR**	**(95% CI)**	***p***
Intracranial haemorrhage	0.625	(0.432–0.906)	0.013
Abdominal bleeding	1.338	(0.24–7.471)	0.740
Thoracic bleeding	0.599	(0.069–5.174)	0.641
Arterial soft tissue haematoma	2.316	(0.575–9.334)	0.238
Venous soft tissue haematoma	0.549	(0.257–1.172)	0.121
Epistaxis	2.316	(0.575–9.334)	0.238
ICU admission	0.958	(0.719–1.276)	0.767
In-hospital mortality	1.085	(0.656–1.796)	0.751
**Outcome**	**GMR**	**(95% CI)**	***p***
LOS hospital [days]	0.987	(0.822–1.186)	0.890

Abbreviation: *DOAC* Direct oral anticoagulants, *GMR* Geometric mean ratio, *ICU* Intensive care unit, *LOS* Length of stay, *OR* Odds ratio

## Discussion

The study compared the proportional incidence of bleeding and other clinical and procedural outcomes in elderly patients on anticoagulation therapy with either VKA or DOAC, after ED presentation due to falls from standing.

In the group taking DOAC, there was a lower proportional incidence of all bleeding types, especially intracranial haemorrhage. This is in line with other studies, that have shown no inferiority and even lower incidences of major life threatening bleeding in patients on long-term DOAC therapy than with VKA [12, 26–30]. A prospective, multicentre study in the Unites States compared the outcome after trauma of patients on different anticoagulation and anti-platelet regimens. Patients on DOAC had a significantly lower rate of intracranial haemorrhage than with VKA, but no difference in short-term mortality [31]. Even though the median age in most studies was above 65 years, to the best of our knowledge, no study has compared the incidence of overall bleeding and other clinical or procedural outcomes after falls from standing specifically in elderly patients. This is particularly important, as this group not only exhibits a higher incidence of atrial fibrillation or post thromboembolic disease than younger patients – both indications for long-term oral anticoagulation –, but also between falls and other comorbidities, such as chronic renal or liver insufficiency, peptic ulcers or multifactorial frailty.

These factors can make a decision to begin with anticoagulation therapy very challenging [32]. Thus, many of these patients remain untreated, despite the indication for anticoagulation. They are then at increased risk of thromboembolic events, especially ischemic stroke, accompanied by morbidity and mortality [15–17, 33]. If in patients at high risk of falling one of the treatment options can be demonstrated to be associated with fewer complications, this could not only reduce complications in treated patients – both directly and indirectly, by reducing the number of untreated patients.

We have replicated the finding of other studies that showed no differences between the two study groups in clinical or other procedural outcomes [34, 35]. This stands in contrast to other studies, that have shown lower mortality in patients under DOAC [20, 34]. Feeney et al. showed that the prognosis of anticoagulated patients who had suffered blunt traumatic intracranial haemorrhage after a fall depends on the type of anticoagulation; patients under DOAC therapy exhibited lower mortality and fewer interventions, irrespective of the severity of the injury and comorbidities [34]. Although there was no age restriction, the mean age in this study cohort was 75 years. Increased mortality in anti-coagulated patients under VKA has also been recently reported for elderly (≥60 years of age) patients treated at an ICU after traumatic brain injury – irrespective of the trauma mechanism [27]. This is an interesting finding, as reversal agents are more often administered to VKA than to DOAC patients after trauma [27, 36].

### Limitations

Our study has several limitations: Although we have made every effort to ensure the best possible data quality, documentation errors cannot be ruled out for any retrospective evaluation. Nevertheless, documentation error is assumed to be equally distributed in all included groups and should therefore not compromise the findings of this study. This is particular the case for the diagnosis bias of bleeding complications. The physician on duty decided on the diagnostic pathway. Such a diagnosis bias is thought to introduce a non-differential misclassification, i.e. equally distributed between DOAC and VKA patients, and should not bias the obtained effect sizes.

Although multivariable analysis and sensitivity analyses were used to take confounding into account, known and unknown confounders might have been missed.

As this was a single centre evaluation at a university hospital, our results may not be representative of other populations.

Adherence to anticoagulation therapy could not be assured in the DOAC group, as – in contrast to the VKA group – the INR is not a marker of the anticoagulation effect, Moreover, the concentration of DOAC is not routinely determined. This could be of particular importance, as diseases of the elderly such as renal insufficiency lead to increased serum levels of DOAC [37], although the correlation with the incidence of bleeding is not yet well understood. However, this lack of a surrogate marker for adherence is counterbalanced by one of the greatest strengths of this study, its real-life design.

Additionally, there might be a selection bias, as both fatal bleeding as well as minor bleeding that did not need ED presentation might not have been included in the analysis. While some might argue that minor bleeding is not of great interest, patients with fatal bleeding that might have occurred on site after the fall might not have been admitted to the ED, and then excluded from the analysis. However, the expected number of immediate deaths caused by haemorrhage after falls from standing is low [38] and, in 95% of all emergency calls, the emergency medical service in Bern arrives at the site of an accident within 15 min [39].

The total number of person-years under anticoagulation is not known, so that only the proportional incidence could be studied. Conclusions therefore can only be drawn with caution.

Longitudinal, prospective, population-based cohort studies are needed to allow the conclusion that – in frail elderly patients who are at risk of future falls and with an indication for anticoagulation – DOACs should be favoured over VKAs in order to reduce the incidence of bleeding complications.

## Conclusion

Elderly, anticoagulated patients treated with DOAC at the ED after a fall from standing have a reduced proportional incidence of overall bleedings and intracranial haemorrhage than elderly patients on VKA.

## Supplementary Information

**Additional file 1: Supplement 1.** Information on laboratory values and platelet aggregation inhibitor (PAI) intake in the two study groups

## Data Availability

All analysed data can be requested from the corresponding author on reasonable request in accordance with Swiss law.

## Abbreviations

DOAC: Direct oral anticoagulant
ED: Emergency department
ICU: Intensive care unit
LOS: Length of stay
VKA: Vitamin K antagonist
