# Supplementary material for: Falls in ED patients: do elderly patients on direct oral anticoagulants bleed less than those on vitamin K antagonists?
Source: Scand J Trauma Resusc Emerg Med. 2021 Apr 6;29:56. doi: 10.1186/s13049-021-00866-6 (PMC8022425; doi:10.1186/s13049-021-00866-6)
Supplement: Supplementary file 1 — Additional file 1: Supplement 1. Information on laboratory values and platelet aggregation inhibitor (PAI) intake in the two study groups [file 13049_2021_866_MOESM1_ESM.docx]

### Supplement 1. Information on laboratory values and platelet aggregation inhibitor (PAI) intake in the two study groups

|  | **Non-missing values** | | **VKA (n=1021)** | | **DOAC (n=426)** | | **Total (n=1447)** | | **p** |
| --- | --- | --- | --- | --- | --- | --- | --- | --- | --- |
| **Laboratory values** |  |  |  |  |  |  |  |  |  |
| Haemoglobin g/l, [med (iqr)] | 1359 | (93.9) | 125 | (111-138) | 123 | (107-137) | 125 | (110-138) | 0.042 |
| Platelets G/l, [med (iqr)] | 1353 | (93.5) | 204 | (165-252) | 213 | (167-260) | 206 | (166-255) | 0.067 |
| Leucocytes G/l, [med (iqr)] | 1356 | (93.7) | 8.7 | (7-11.1) | 8.8 | (6.8-11) | 8.8 | (6.9-11.1) | 0.934 |
| INR, [med (iqr)] | 1332 | (92.1) | 2.1 | (1.3-2.9) | 1.2 | (1.1-1.3) | 1.6 | (1.1-2.6) | <0.001 |
| Creatinine umol/l, [med (iqr)] | 1349 | (93.2) | 94 | (73-124.5) | 88 | (70-113) | 91 | (72-121) | 0.006 |
| Sodium mmol/l, [med (iqr)] | 1344 | (92.9) | 139 | (136-141) | 139 | (135-141) | 139 | (136-141) | 0.281 |
| Potassium mmol/l, [med (iqr)] | 1346 | (93.0) | 4.1 | (3.8-4.4) | 4.1 | (3.7-4.4) | 4.1 | (3.8-4.4) | 0.331 |
| **PAI, [n (%)]** | 1447 | (100.0) |  |  |  |  |  |  |  |
| None |  |  | 818 | (80.1) | 362 | (85.0) | 1180 | (81.5) |  |
| Aspirin |  |  | 153 | (15.0) | 51 | (12.0) | 204 | (14.1) |  |
| Clopidogrel |  |  | 31 | (3.0) | 9 | (2.1) | 40 | (2.8) |  |
| Aspirin and clopidogrel |  |  | 19 | (1.9) | 3 | (0.7) | 22 | (1.5) |  |
| Ticagrelor |  |  | 0 | (0.0) | 1 | (0.2) | 1 | (0.1) | 0.067 |

**Abbreviation:** DOAC: direct oral anticoagulant, iqr: interquartile range, med: median, PAI, platelet aggregation inhibitor, VKA: vitamin K antagonist.
